# Walking behaviours from the 1965–2003 American Heritage Time Use Study (AHTUS)

**DOI:** 10.1186/1479-5868-4-45

**Published:** 2007-09-27

**Authors:** Catrine Tudor-Locke, Hidde P van der Ploeg, Heather R Bowles, Michael Bittman, Kimberly Fisher, Dafna Merom, Jonathan Gershuny, Adrian Bauman, Muriel Egerton

**Affiliations:** 1 Walking Research Laboratory, Department of Exercise and Wellness, Arizona State University, Mesa, Arizona, 85212, USA; 2 The Centre for Physical Activity and Health, University of Sydney, Sydney, New South Wales, Australia; 3 School of Social Science, University of New England, Armidale, Australia; 4 Centre for Time Use Research, University of Oxford, Oxford, UK

## Abstract

**Background:**

The American Heritage Time Use Study (AHTUS) represents a harmonised historical data file of time use by adults, amalgamating surveys collected in 1965–66, 1975–76, 1985, 1992–94, and 2003. The objectives of time-use studies have ranged from evaluating household and other unpaid production of goods and services, to monitoring of media use, to comparing lifestyles of more and less privileged social groups, or to tracking broad shifts in social behaviour. The purpose of this paper is to describe the process and utility of identifying and compiling data from the AHTUS to describe a range of walking behaviours collected using time-use survey methods over almost 40 years in the USA.

**Methods:**

This is a secondary data analysis of an existing amalgamated data set. Noting source survey-specific limitations in comparability of design, we determined age-standardized participation (and associated durations) in any walking, walking for exercise, walking for transport, walking the dog, sports/exercise (excluding walking), and all physical activity for those survey years for which sufficient relevant data details were available.

**Results:**

Data processing revealed inconsistencies in instrument administration, coding various types of walking and in prompting other sport/exercise across surveys. Thus for the entire period, application of inferential statistics to determine trend for a range of walking behaviours could not be done with confidence. Focusing on the two most comparable survey years, 1985 and 2003, it appears that walking for exercise in America has increased in popularity on any given day (from 2.9 to 5.4% of adults) and accumulated duration amongst those who walk for exercise (from 30 to 45 mins/day). Dog walking has decreased in popularity over the same time period (from 9.4 to 2.6%). Associated duration amongst dog walkers was stable at 30 mins/day.

**Conclusion:**

The noted and sometimes substantial differences in methods between the various survey administrations preclude stringent interpretation of these trends in walking behaviours and the use of conventional application of inferential statistics to evaluate significance of time trends. Although the AHTUS offers the most comprehensive attempt at harmonization yet undertaken with these individual time-use surveys, we found that any noted cross-time changes in walking and physical activity behaviour are not easily interpreted in terms of conventional epidemiological approaches and could be true changes, artefact related to instrument and method changes, or both. Public health utilization of the AHTUS, could be enhanced with greater attention to methodological issues known to influence estimation of physical activity behaviour in population. This could be achieved with cross-disciplinary collaboration between groups of experts in the various stages of these surveys.

## Background

The benefits of a physically active lifestyle are diverse and well known yet surveillance systems continue to track low levels of prevalence of this health-promoting behaviour [1]. Of all types of physical activity (PA), walking is both most commonly encouraged [2] and most commonly reported [3,4], especially in the form of walking for exercise. Walking for exercise has also been described in terms of "leisure-time" walking and is reasonably similarly distributed across social groups and by gender and age [3,4]. Regardless of the applied label, this mode of walking characteristically represents that which is typically undertaken for its own sake (i.e., purposeful) and directly to improve some aspect of health. The Behavioral Risk Factor Surveillance System (a large, telephone-based survey) has been used to examine walking for exercise (their specific terminology is leisure-time walking) trends among American adults [5]. Prevalence of (any) walking for exercise increased absolutely 3.8% (from 26.2% to 30.1%) in men and 6.6% (from 40.4% to 46.9%) in women between 1987 and 2000. The median frequency of walking for exercise remained stable at 2.9 times/week and 30 minutes/session; public health guidelines promote at least 5 days/week (of any PA of at least moderate intensity, including walking) at this duration.

Another mode of walking, walking for transportation, has more recently entered the public health arena as a plausibly important contributory source of PA that can be achieved easily within the Westernized and accepted culture of multi-tasking. Walking for transportation appears to meet the minimal requirements for health-enhancing PA: bouts are of sufficient duration [6] known to elicit cardirorespiratory benefits [7-9]; self-selected walking paces appear to be naturally of moderate intensity [10,11], and walking for transportation appears to be more commonly (i.e., a greater proportion of the population on any given day and more days of the week) performed than walking for exercise [6]. Further, empirical evidence of the health benefits associated explicitly with walking for transportation is accumulating [12-14]. In the USA, Healthy People 2010 Objective 22–14 calls for increasing the proportion of (short) trips made by walking [15]. Specifically, the 2010 target for adults is 25% of all trips ≤ 1 mile and for children and adolescents it is 50% of trips to school ≤ 1 mile. A limited examination of walking for transportation trends (amalgamating 2001 National Household Travel Survey and the 1995 Nationwide Personal Transportation Survey) suggests that American adults walked more short trips in 2001 (21.2%) compared to 1995 (16.7%); a similar result was found for youth (35.9% in 2001 vs. 31.3% in 1995) [16]. Differences in methods between the two survey administrations likely temper interpretation of time trends.

Few rigorous data exist to directly compare trends in walking for exercise and transportation to general sports/exercise participation or other potential sources of health-promoting walking such as walking the dog [17]. Recently, however, a harmonised historical data file of time use by adults in the USA., covering surveys collected in 1965–66, 1975–76, 1985, 1992–94, and 2003, has been made freely available to the research community at www.timeuse.org/AHTUS. This data set, known as the American Heritage Time Use Study (AHTUS), was developed for the Yale University Program on Non-Market Accounts with funding from the Glaser Progress Foundation. Time-diary methods have an extensive global history in the social sciences [18] and are distinctive because they collect a complete log of the respondents' (or diarists', applying time use terminology conventions) activities over a 24-hour period. Time-use data typically capture starting and ending times, diarists' primary activity, 'what else' they were doing at the same time (i.e., secondary activity, although this is not uniformly captured in all surveys), the location of the activity (e.g., outside), and the presence of others during the activity.

Collecting the time allocated to walking (regardless of purpose) by using time-diaries has some clear advantages over conventional activity surveys relying on respondents' self-report of targeted behaviours. Firstly, the time-use surveys require diarists to report all their activities in their own words for a full 24-hour period. This yields, among other things, a comprehensive picture of walking as a deliberate form of exercise, as a mode of transport, or even as a form of pet care within the context of daily life and competing activities (including other sports/exercises). Secondly, precisely because diarists are asked for a comprehensive account of activities, their responses are far less affected by well-known social desirability biases more commonly associated with self-reported 'typical' behaviours [19]. Further, faking a time-diary entry requires careful consideration of the plausibility of what was recorded before and after the fallacious activity, and deliberately falsifying activity accounts takes more effort than recording legitimate accounts (except in the rare case of very low episode diaries), giving respondents an additional internal incentive to recall their actual activities [20]. Although Levin et al. [21] have shown that intra-individual variability is sufficient to warrant multiple repetitions of 48-hour physical activity records to achieve reliable estimates of habitual physical activity, time use diaries differ in that: 1) they do not focus specifically on physical activity but may be mined for physical activity in the context of all activities undertaken; and 2) they are not intended to be used to interpret individual behaviour but are rather to be used to identify population (or subgroup) time use patterns "on any given day." Time use researchers recognize that optimal diary duration (or length of coverage) varies across different types of activity, as a function of the "cycle" time between repetitions of the target event. For example, estimations of meal times (or repetitive habitual excursions of walking for transportation), require only a short diary duration. In contrast, occasional performance of recreational hikes or walking around the zoo, for example, logically require longer durations. Regardless, a succession of validation studies has confirmed that time allocation data collected by time-diaries are more accurate than the estimates derived from more general queries of time spent in a single or a group of listed activities [19,20,22].

As social surveys, the primary purposes of time-use studies has ranged from evaluating household and other unpaid production of goods and services, to monitoring of media use, to comparing lifestyles of more and less privileged social groups, or to tracking broad shifts in social behaviour [23]. These data also represent a rich and largely untapped resource for exploring health-related behaviours, including participation in sports/exercise and different sources of walking. Previously, we took advantage of the 1997 Australian Bureau of Statistics (ABS) Time Use Survey to describe nationally representative patterns of walking for transport and for exercise in Australian adults [6]. The harmonized American data set further provides an opportunity to look into historic trends in these behaviours among Americans, to the extent to which component surveys comparatively captured and coded these specific variables. Therefore, the purpose of this paper is to describe the process and utility of using the AHTUS to describe any walking, but specifically walking for exercise and transportation as well as dog walking and sports/exercise participation (i.e., this latter to contextualize the contribution of walking to PA), and all PA collected using time-use survey methods over almost 40 years in the USA. We discuss the extent to which these variables can be confidently compared across surveys and the limitations confronted.

## Methods

### Data source

The AHTUS unifies data from five separate major representative national probability sample American time-use surveys to produce an individual-level dataset spanning 38 years from 1965 through 2003 and representing over 36,500 diaries from people aged 18 and older. These five constituent datasets are: 1) Americans' Use of Time, part of the Multinational Comparative Time-Budget Research Project, Jackson and national USA samples (1965–66); 2) the University of Michigan Time Use in Economic and Social Accounts (1975–76); 3) the University of Michigan American's Use of Time (1985); 4) US. Environmental Protection Agency National Human Activity Pattern Survey (NHAPS) (1992–94); and, 5) the Bureau of Labor Statistics American Time Use Survey (2003). The methodological characteristics of these surveys and the specific approaches used in harmonizing these studies are detailed extensively in the associated on-line documentation but are also summarized briefly in Table 1, having adapted the table from Fisher et al. [24].

**Table 1:** Comparative summary of time use surveys constituting the AHTUS with a focus on walking behaviours

Year(s)	1965–1966	1975–1976	1985	1992–1994	2003
Name	Americans' Use of Time (part of the Multinational Comparative Time-Budget Research Project)	Time Use in Economic and Social Accounts	American's Use of Time	US. Environmental Protection Agency National Human Activity Pattern Survey	American Time Use Survey
Organization	Survey Research Center, University of Michigan	Survey Research Center, University of Michigan	Survey Research Center, University of Maryland	Survey Research Center, University of Maryland	United States Bureau of Labor Statistics and United States Census Bureau
Sample	Jackson 759 diaries National 1262 diaries	4584 diaries (main respondent only)	2636 diaries	7514 diaries	19,663 diaries
Age range	19–65 (some up to 69)	18+	12+ (18+ in AHTUS)	0+ (18+ in AHTUS)	15+ (18+ in AHTUS)
Response rate	82% Jackson, 74% National	72% first wave, 45% did all 4 waves	55% overall, 51% for mail back sample	63%	58%
Collection method	Self-completion with guidance from interviewer	Self-report waves 1&4, phone waves 2 &3*	3 samples: CATI, self-completion, personal interview (only self-completed in AHTUS)	CATI	CATI
Walking variables captured	Any walking Walking for exercise	Any walking Walking for exercise	Any walking Walking for exercise Walking for transport Walking the dog	Any walking Walking for transport Walking the dog (Walking for exercise was captured under either Walking for Transport or, for this year only, Sports &Exercise)	Any walking Walking for exercise Walking for transport Walking the dog
Other PA variables	Sports & exercise** Total PA***	Sports & exercise** Total PA***	Sports & exercise** Total PA***	Sports & exercise Total PA***	Sports & exercise** Total PA***

Adapted from Fisher et al.[24] *for these analyses we randomly sampled one wave for each participant of the 1975 survey; **walking for exercise not included; ***all walking and sports & exercise considered, no double-counting

### Data treatment

The AHTUS includes 91 independent main activities in the harmonized data sets. The specific walking and other PA variables captured for each survey year are also catalogued in Table 1. Where walking was indicated as the main activity, this was designated as walking for exercise. The 1992–1994 interviewer and coding instructions did not facilitate the separate coding of walking in a way that is comparable with the other years of data. Specifically, the 1992–1994 survey did not separately code walking for exercise; it was encapsulated under walking for transport but also under a combined category of sports/exercise. These could not be disentangled and represent a comparability issue that is characteristic of such large, amalgamated independent data sources spanning many years.

When the main activity was recorded as "transportation" the harmonized dataset also included codes for mode of transport, and walking for transportation was identified. However, mode of transport was not recorded in the 1965 and 1975 surveys. Therefore, walking for transportation was captured for survey periods 1985, 1992–1994, and 2003 only. In the 1985 data set walking for transport was both included in walking for exercise as well as reported separately; to get at the correct value for walking for exercise, walking for transport was therefore subtracted from the total value.

If "pet care" was identified as the main activity and the activity was performed outside for a minimum duration of 10 minutes this was designated as time spent walking the dog. Walking the dog was only reportable in 1985, 1992–1994, and 2003.

Sport/exercise participation was defined as recording "sports/exercise," "cycling," "outdoor recreation," or "physical activity/sports with child" as a main activity. Walking for exercise was excluded from this definition of sport/exercise participation to avoid double-counting of this activity. An exception to this rule, as alluded to above, was that encountered with the 1992–1994 survey; walking for exercise could have been captured under sports/exercise, limiting comparability of this particular survey year's sport/exercise participation to other years. Further, the 1965 dataset did not include codes for cycling or outdoor recreation as main activities. Although the other surveys included codes for cycling and outdoor recreation as main activities, the 1992–94 survey sample did not include any records where cycling was a main activity, and the 2003 sample did not include any records where outdoor recreation was identified as a main activity. It is possible that some respondents did engage in these activities during these surveys but their data were not separated in a manner necessary to capture this level of detail during the coding process.

The AHTUS variable representing activity time in minutes was used to summarize accumulated minutes in the targeted activities. Where minutes were > 0, diarists were labeled 'doers' (identifying a participant sub-sample). As we have previously noted [6] this terminology (i.e., doers) is widely accepted in time-use research as referring to the participant sub-sample performing the said activity as originally set out by the foundational work of Szalai et al. [18]. We believe it is appropriate to continue use of this common terminology in the general study of PA and public health. The prevalence (and 95% CI) of the different activities is presented, as well as mean and 95% CI (and additionally median, and 25^th ^and 75^th ^percentiles of distribution) of time spent in these activities by the doer sub-samples. Prevalence of achievement of ≥ 30 minutes of at least moderate intensity PA "on any given day" was evaluated by considering accumulated bouts of ≥ 10 minutes of any walking, other sports/exercise (excluding walking), and for all PA (sports/exercise/all walking). Prevalence estimates were age-standardized to the 2000 US. census population using age groups 18–24 years, 25–34 years, 35–44 years, 45–64 years, and 65 years or older.

We did not include secondary (or simultaneous) activities in this analysis for two reasons. First, the two most recent surveys (1992–94 and 2003) did not collect secondary activity, though the other surveys did collect this information. We opted for the most comparable information on walking across surveys. Second, very little PA is recorded as a secondary activity in time-use surveys, so potential losses would be minimal (unpublished data, K. Fisher). We investigated this further: only 3 episodes of secondary walking were recorded in the 1965–1966 as well as in the 1985 surveys. Thirteen such episodes were recorded in the 1975–1976 survey – making a total of 19 potentially affected episodes. In one case, the main activity also was recorded as walking, and in another the main activity was pet care, but the treatment of pet care described above would still have captured this activity. This leaves only 17 episodes lost as a consequence of our decision, though as these episodes span a range of activities from adult care to work breaks, it is not clear whether more of such episodes might have appeared in the more recent surveys had they also collected secondary activity.

As a result of the multiple issues identified above, we made a concerted decision to focus on the description of the targeted PA variables (and their apparent changes between survey administrations) and to forego using inferential statistics to make judgments about significance of observed differences.

## Results

Speaking to the primary purpose (i.e., process and utility of identifying and compiling data from the AHTUS), the comparative summary of methods displayed in Table 1 outline the challenges undertaken herein (including the different ways in which variables were captured and expressed) and the missing data displayed in Tables 2 and 3 hamper conclusions about changes in physical activity trends. Although the AHTUS offers the most comprehensive attempt at harmonization yet undertaken with these individual time-use surveys, any noted cross-time changes are not easily interpreted in terms of conventional epidemiological approaches and could be true changes, artefact related to instrument and method changes, or both. Therefore any descriptive data presented must to duly interpreted with caution.

**Table 2:** Prevalence*of walking behaviours, sports and exercise, and total physical activity

	**Proportion of doers**				
	
	**1965**	**1975**	**1985**	**1992–1994**	**2003**
	**(N = 1651)**	**(N = 1555)**	**(N = 2923)**	**(N = 7527)**	**(N = 19714)**
	
**Physical activity variable**	**n**	**n**	**n**	**n**	**n**
	**%**	**%**	**%**	**%**	**%**
	**(95% CI)**	**(95% CI)**	**(95% CI)**	**(95% CI)**	**(95% CI)**
Any walking	39	57	443	1577	3720
	2.4	3.7	15.2	21.0	18.9
	(1.6; 3.1)	(2.7; 4.6)	(13.9; 16.5)	(20.0; 21.9)	(18.3; 19.4)
Walking for exercise	39	57	85	NA	1073
	2.4	3.7	2.9		5.4
	(1.6; 3.1)	(2.7; 4.6)	(2.3; 3.5)		(5.1; 5.8)
Walking for transport	NA	NA	105	1244	2845
			3.6	16.5	14.4
			(2.9; 4.3)	(15.7; 17.4)	(13.9; 14.9)
Walking the dog	NA	NA	275	408	506
			9.4	5.4	2.6
			(8.3; 10.5)	(4.9; 5.9)	(2.3; 2.8)
Sports/exercise**	97	188	501	1438	2427
	5.9	12.1	17.1	19.1	12.3
	(4.7; 7.0)	(10.5; 13.7)	(15.8; 18.5)	(18.2; 20.0)	(11.9; 12.8)
Total physical activity***	134	237	850	2602	5540
	8.1	15.2	29.1	34.6	28.1
	(6.8; 9.4)	(13.5; 17.0)	(27.4; 30.7)	(33.5; 35.6)	(27.5; 28.7)

NA = data not available; *Prevalence estimates age-standardized to the 2000 US. census population using age groups 18–24 years, 25–34 years, 35–44 years, 45–64 years, and 65 years or older; **walking for exercise not included, with the exception of 1992–1994 which might have contained walking for exercise; ***all walking and sports/exercise considered, no double-counting

**Table 3:** Doer sub-sample accumulated daily minutes in walking behaviours, sports and exercise, and total physical activity

	**Minutes doers spent in activity**				
	
	**1965**	**1975**	**1985**	**1992–1994**	**2003**
	
**Physical activity variable**	**mean (95% CI)**	**mean (95% CI)**	**mean (95% CI)**	**mean (95% CI)**	**mean (95% CI)**
	**median (P**_25;_**P**_75_**)**	**median (P**_25;_**P**_75_**)**	**median (P**_25;_**P**_75_**)**	**median (P**_25;_**P**_75_**)**	**median (P**_25;_**P**_75_**)**
Any walking	46.2	51.9	44.8	40.2	44.9
	(36.7; 55.7)	(37.2; 66.6)	(40.8; 48.8)	(37.4; 43.1) 20	(43.0; 46.7)
	43	30	30	(10; 50)	30
	(23; 60)	(30; 60)	(20; 60)		(10; 60)
Walking for exercise	46.2	51.9	42.0	NA	52.4
	(36.7; 55.7)	(37.2; 66.6)	(37.1; 47.0)		(49.6; 55.2)
	43	30	30		45
	(23; 60)	(30; 60)	(29; 60)		(30; 60)
Walking for transport	NA	NA	53.8	32.7	31.4
			(44.2; 63.5)	(30.2; 35.2)	(29.9; 32.8)
			45	20	20
			(30; 60)	(8; 45)	(6; 42)
Walking the dog	NA	NA	38.8	55.9	42.5
			(34.1; 43.6)	(48.4; 63.4)	(39.4; 45.5)
			30	30	30
			(15; 50)	(20; 60)	(20; 60)
Sports/exercise **	95.1	108.3	87.7	122.1	89.6
	(80.0; 110.2)	(94.6; 122.0)	(81.1; 94.3)	(116.0; 128.2)	(86.5; 92.6)
	90	85	60	80	60
	(25; 150)	(40; 143)	(30; 120)	(45; 160)	(40; 120)
Total physical activity***	81.8	98.5	75.1	91.9	69.4
	(70.0; 93.6)	(86.7; 110.3)	(70.3; 79.9)	(87.7; 96.0)	(67.4; 71.3)
	60	60	55	60	50
	(25; 130)	(30; 126)	(30; 97)	(20; 120)	(20; 90)

*estimates age-standardized to the 2000 US. census population using age groups 18–24 years, 25–34 years, 35–44 years, 45–64 years, and 65 years or older; **walking for exercise not included, with the exception of 1992–1994 which might have contained walking for exercise; ***all walking and sports & exercise considered, no double-counting

That being said, the age-standardized prevalence of any walking, walking for exercise, walking for transport, walking the dog, sports/exercise (excluding walking), and for all PA are presented in Table 2. As indicated earlier, not all of these variables are captured for all surveys; the most similarly complete data sets are 1985 and 2003, and the latter is superior to all previous years in terms of data quality. Directly interpreted, the prevalence of any walking ranged from a low of 2.4% in 1965 to highs of 18.9% in 2003 and 21% in 1992–1994. The prevalence of walking for exercise in 2003 appears (interpreting CI for overlap) higher than all previous years. Walking for transport has increased from the first year (1985) in which it was captured; 1992–1994 shows a higher prevalence of walking for transport compared to either 1985 or 2003. Walking the dog displayed a steady decrease in prevalence over the three years it was captured. Sports/exercise increased from 1965 to 1975 and again in 1985, where it remained stable through 1992–1994, and decreased in 2003. The prevalence of total physical activity increased every survey year until 2003, which displayed a drop to 1985 levels.

Table 3 shows the mean and median minutes (with associated dispersion statistics) spent in these different activities by the doer sub-sample. Interpreting the overlapping 25^th ^and 75^th ^percentiles associated with the median values for each survey year, time spent in any walking (medians ranging from 20–43 minutes), or more specifically, walking for exercise (medians ranging from 30–45 minutes) appears stable among doers across survey years. Looking at the means and their 95% CI, doers in 2003 are averaging a longer duration of walking for exercise (i.e., 10–15 minutes longer on any given day) compared to 1985. Although median values of time spent walking the dog appeared stable across the three years captured, there was an increase in mean time spent by doers in this activity from 1985 to 1992–1994, but stable thereafter. Median time spent in sports/exercise range from a high of 90 minutes in 1965 to a low of 60 minutes in 2003. A drop in mean time spent by doers in sports/exercise from 1975 to 1985, increased to a high in 1992–1994 (note sports/exercise in this single survey year also included walking for exercise that could not be extracted), and decreased again in 2003 (although to a comparable level as 1985). Median total physical activity amongst doers ranged from a low of 50 minutes in 2003 to a high of 60 minutes in 1965, 1975, and 1992–1994. Mean time spent in total physical activity by doers was lower in 1985 compared to previous years, increased again in 1992–1994, and decreased in 2003 to a level not apparently different from 1985.

## Discussion

The current growing focus on a broad range of walking behaviours as a source of healthful physical activity is the product of a number of related factors including: 1) the US. Surgeon General endorsed public health guidelines that promote daily activity that is of at least 30 minutes in duration and of at least moderate intensity (equivalent to brisk walking) [25]; 2) the proportion of American adults who achieve recommended levels of physical activity has continued to be low [26]; 3) high levels of attrition from structured exercise programs is a well-known phenomenon [27]; 4) research on the benefits of active living vs. prescriptive exercise expanded beliefs about dose-response relationships [28]; 5) the explosion of research related to the impact of the built environment on physical activity continues to fuel interest in transportation-related and lifestyle-related walking [29]; and, 6) the persisting popularity of objective monitoring using body worn instruments (including accelerometers and pedometers) has greatly advanced researchers', practitioners', and lay peoples' interest in and ability to quantify physical activity volumes [30]. Against this context, and in the face of few complete and consistent data sources, it is rational to pursue the feasibility of amalgamation of existing data sets in an attempt to establish foundational data and begin to examine cross-time patterns.

Previously, an amalgamation of the 2001 National Household Travel Survey and the 1995 Nationwide Personal Transportation Survey, also despite methodological differences in survey administration, has been used to examine differences in walking behaviours over a 6-year time period [16]. Therefore the opportunity to closely examine walking behaviours using this time use data source over an extended time period was not to be passed up, that is, a thorough examination of its public health utility in terms of physical activity trends was warranted. However, we must duly conclude that the noted and sometimes substantial differences in methods between the various survey administrations preclude stringent interpretation of these trends in walking behaviours and the use of conventional application of inferential statistics, including multiple linear regression, to evaluate significance of time trends.

With the strong caveat above, we focused our exploration of walking behaviours within the AHTUS to walking for exercise, for transportation, and for the purpose of pet care (i.e., walking the dog). Considering any walking (of those identified), 1965 and 1975 showed very low prevalence (2.4 to 3.7%) but jumped to much higher levels (15–21%) in later years probably because walking for transport and dog walking were counted. Focusing on the two most comparable survey years, 1985 and 2003, it appears that walking for exercise in America has increased in popularity on any given day (from 2.9 to 5.4% of adults) and accumulated duration amongst doers (from 30 to 45 mins/day). Dog walking, on the other hand has decreased in popularity over the same time period (from 9.4 to 2.6%). Those diarists who walked dogs in either 1985 or 2003 tended to do so for an average of 30 mins/day. Again, with the caveats mentioned above considered, these data do not support a dramatic decrease in walking behaviours over time, calling into question generalized concerns about the related impact of built environment and the role that such behaviours play in the acknowledged obesity epidemic. We must acknowledge that walking may be undertaken in the course of other activities not considered in the AHTUS including work, shopping, sightseeing and other forms of recreation, and chores/errands, to name but few plausible additional sources. Using the Australian Time Use data, we previously showed that the greatest proportion (31.9%) of daily time spent in transportation-related walking is for the purpose of shopping, followed by work (23.3%), social and community interaction (13.1%), and recreation and leisure (10.2%) [6]. A similar analysis was not possible with the AHTUS where we were limited to the 91 specific activities harmonized over the survey years.

As the only other published time use study of walking behaviours, however, the 1997 Australian Time Use data provide an important source of comparison. For example, the Australian survey indicated a higher prevalence of walking for transport vs. walking for exercise (20 vs. 9%) in 1997. In the two most comparable AHTUS years, 1985 and 2003, the prevalence of walking for transport was 3.6 and 14.4% respectively, and the prevalence of walking for exercise was 2.9 and 5.4% respectively. Both types of American walking behaviours in both years were less common than that reported by Australians in 1997. In terms of cumulative duration, Australian doers accumulated a median of 28 mins/day of walking for transport and 56 mins/day walking for exercise in 1997. In 1985, American doers accumulated a median of 45 mins/day of walking for transport and 30 mins/day walking for exercise. In, 2003, the comparable values were 20 minutes/day and 45 minutes/day. Stated simply: 1) although walking for transportation was of a longer accumulated duration on any given day for American doers in 1985 compared to Australian doers in 1997, the accumulated duration was shorter in 2003, and, 2) walking for exercise was of a shorter accumulated duration on any given day for Americans doers for both years compared to Australian doers in 1997. It must be noted, however, that although the Australian Time Use survey does consider two days of individual data compared to the AHTUS surveys which consider only a single day, data analysis checks have previously validated the use of the person-day in the Australian data for interpreting prevalence of participation in such activities [6].

## Conclusion

The expressed purpose of this paper was to describe the process and utility of using the opportunity presented by the AHTUS to capture and describe changes in walking behaviours across almost 40 years in the USA. Although the AHTUS data are a rich resource, do provide a good measure of social activities, and have been successfully used in other non-walking related studies [19], the noted limitations confronted by the cross-disciplinary team of experts assembled here challenge any firm conclusions about physical activity trends. However, we anticipate that planned consistent administration of the American Time Use Survey (with data currently available from 2003, 2004 and 2005; www.bls.gov/tus/) will be more useful in terms of providing useful public health related trend information. This study provides an important blueprint for guiding multi-disciplinary future research that might enhance the public health utility to time use studies in terms of physical activity behaviours.

## Competing interests

The author(s) declare that they have no competing interests.

## Authors' contributions

CTL, MB, HPvdP, and HRB conceived and designed the study.

HPvdP led the analysis with critical inputs from HRB, KF, MB, and AB

KF, ME, and JG provide critical direction in use of the AHTUS and assisted with interpretation in the context of time-use methods

HRB, DM, and AB provided additional interpretation in the context of epidemiologic methods

CTL led the primary writing group of HPvdP, MB, HRB, KF, DM, and AB

All authors read and approved the final manuscript.
